# Manual Gestures Modulate Early Neural Responses in Loudness Perception

**DOI:** 10.3389/fnins.2021.634967

**Published:** 2021-09-01

**Authors:** Jiaqiu Sun, Ziqing Wang, Xing Tian

**Affiliations:** ^1^Division of Arts and Sciences, New York University Shanghai, Shanghai, China; ^2^NYU-ECNU Institute of Brain and Cognitive Science, New York University Shanghai, Shanghai, China; ^3^Shanghai Key Laboratory of Brain Functional Genomics, Ministry of Education, School of Psychology and Cognitive Science, East China Normal University, Shanghai, China

**Keywords:** multisensory integration, cross-modal modulation, audiovisual, manual gesture, motion perception, action, loudness perception

## Abstract

How different sensory modalities interact to shape perception is a fundamental question in cognitive neuroscience. Previous studies in audiovisual interaction have focused on abstract levels such as categorical representation (e.g., McGurk effect). It is unclear whether the cross-modal modulation can extend to low-level perceptual attributes. This study used motional manual gestures to test whether and how the loudness perception can be modulated by visual-motion information. Specifically, we implemented a novel paradigm in which participants compared the loudness of two consecutive sounds whose intensity changes around the just noticeable difference (JND), with manual gestures concurrently presented with the second sound. In two behavioral experiments and two EEG experiments, we investigated our hypothesis that the visual-motor information in gestures would modulate loudness perception. Behavioral results showed that the gestural information biased the judgment of loudness. More importantly, the EEG results demonstrated that early auditory responses around 100 ms after sound onset (N100) were modulated by the gestures. These consistent results in four behavioral and EEG experiments suggest that visual-motor processing can integrate with auditory processing at an early perceptual stage to shape the perception of a low-level perceptual attribute such as loudness, at least under challenging listening conditions.

## Introduction

Imagine that you are boasting about the size of the fish you caught last weekend to your friend. You would probably raise your voice volume when you say the word “big,” and at the same time move your hands away from each other. The iconic gestures in this example not only represent the size of the fish visually but also parallel the volume of your voice. Let’s go a bit further. Suppose that two utterances have the same intensity; if a gesture accompanies one but not the other sound, would you perceive one sound as quieter or louder than the other sound? In general, whether and how the informational contents in one modality penetrate the processing in another modality is a fundamental question for understanding the nature of human perception.

Multisensory integration has been extensively documented (Calvert et al., 2004; Ghazanfar and Schroeder, 2006; Stein and Stanford, 2008). In the domain of multisensory audiovisual interaction, most studies explored the cross-modal effects in ecologically valid connections. For example, the McGurk effect (McGurk and MacDonald, 1976) is established by naturally linked speech categorical representations in the visual and auditory domain (Möttönen et al., 2002; Besle et al., 2004; van Wassenhove et al., 2005, 2007; Arnal et al., 2009; Baart et al., 2014). The ventriloquist effect is based on a high probability in the natural world that the source of visual and auditory information comes from a common identity and location (Howard and Templeton, 1966; Alais and Burr, 2004; Bonath et al., 2007; Alais et al., 2010). However, the boundary and efficacy of cross-modality modulation effects have not been thoroughly explored. For example, it has been extensively demonstrated that gestures and language processing are linked (Krauss, 1998; Arbib et al., 2008; Goldin-Meadow and Alibali, 2013). Most studies revealed this cross-modal connection at higher levels such as semantic, lexical, and phonological levels. Studies on cross-modal interaction occurring for low-level perceptual attributes were relatively rare, such as loudness in auditory perception and distance in visual perception. Compared with other auditory perceptual attributes such as phonetic, phonological, and prosodic features of speech sound, the perceived loudness is at a lower level in the hierarchy of auditory and speech processing.

Recent studies of audiovisual integration using gestures can provide some hints. Gestures can influence auditory perception *via* the linked speech categorical representations at the semantic and phonological levels (Kelly et al., 2004; Özyürek et al., 2007; Willems et al., 2007). For example, gestures (either semantically matching or mismatching) interacted with the N1–P2 auditory responses of words (Kelly et al., 2004). Gestures such as beat (Hubbard et al., 2009) and clapping (Stekelenburg and Vroomen, 2012; van Laarhoven et al., 2017) also influence auditory processing *via* a spatial–temporal contingency. Such modulation resulted from the expected frequency of an acoustic event predicted by the gesture (van Laarhoven et al., 2017). Recently, the basis of cross-modal connections has extended to more basic features such as direction. For example, manual directional gestures can facilitate learning lexical tones in Mandarin Chinese (Zhen et al., 2019). All these results suggest that gestures and acoustic features may share overlapped or transformable representations that would enable across-modal integration for low-level perceptual attributes that do not necessarily link in two modalities. One more interesting phenomenon is that when human participants instinctively made gestures during listening to music, the position of gesture positively correlated with the intensity of the sound (Caramiaux et al., 2010). Will the universal dimension of magnitude, the lowest level perceptual attribute in the perception of all modalities, serves as a connection for multisensory integration in general and a basis for gestural effects on auditory perception in particular?

In this study, we investigated whether and how manual gestures can modulate loudness perception. We developed a new multimodal paradigm in which participants heard the same vowel/a/twice with manual gestures concurrently presented with the second sound. Participants judged the loudness change of the second sound relative to the first sound. We hypothesized that the visual-motion information of gestures would modulate the perceived loudness. To test this hypothesis, we first carried out two behavioral experiments (BE1 and BE2). In BE1, we probe the effects of natural motion gestures on the judgments of loudness changes. To distinguish which features (the distance or the motion) of the gestures influenced the judgments of loudness changes, we carried out BE2 using still images of gestures. We carried out two more EEG experiments (EE1 and EE2) to further investigate whether the effects were perceptual (rather than decisional) in nature by examining the temporal dynamics of the modulation effects. Specifically, we compared the early auditory event-related potential (ERP) responses between conditions of different gestures (EE1) and between trials of different loudness judgment to the same sound (EE2).

In the EEG experiments, we focused on the ERP N100 component that is an early cortical response reflecting (auditory) perceptual analysis (Roberts et al., 2000). The auditory N100 is a fronto-centrally distributed negative wave that is mainly generated in the (primary and associative) auditory cortex (Näätänen and Picton, 1987). Previous studies found that N100 amplitude correlates with perceived loudness. Schmidt et al. (2020) observed that the preceding tone (inducer tone) decreased the perceived loudness of the target tone. Tian et al. (2018) demonstrated that the preceding imagined speech lowered the loudness ratings of the target sound. Both studies showed that the contextual effects on changing loudness perception correlated with the magnitude changes in N1/P2 components in the responses to the target sound. In addition, Lu et al. (1992) observed that the decay rate of N100 amplitude correlated with the decay rate of the loudness perception. Our results suggested that certain visual-motion information in manual gestures modulated the early auditory neural responses (around 110 ms) that corresponded to changes in loudness perception at the just-noticeable difference (JND) threshold.

## Materials and Methods

### Participants

Fifteen young adults (10 females; mean age, 22.1 years; range, 19–25 years) participated in BE1; 12 young adults (7 females; mean age, 22.0 years; range, 20–24 years) participated in BE2; 23 young adults (16 females; mean age, 22.0 years; range, 17–27 years) participated in EE1; 20 young adults (10 females; mean age, 21.8 years; range, 18–25 years) participated in EE2. There was no overlapping of the participants among all four experiments. All participants were native Chinese speakers. They all had normal hearing and normal or corrected-to-normal vision (this was listed in the requirement when recruiting participants, and we also verbally confirmed that). None of them had any neurological deficits (self-reported). They received monetary incentives for their participation. Written informed consents were obtained for all participants before the experiments. The local Research Ethics Committee at NYU Shanghai approved all protocols.

### Stimuli and Trial Procedure

#### Behavioral Experiment 1 (BE1): The Effects of Motional Gestures on the Judgment of Loudness Changes

**FIGURE 1:**
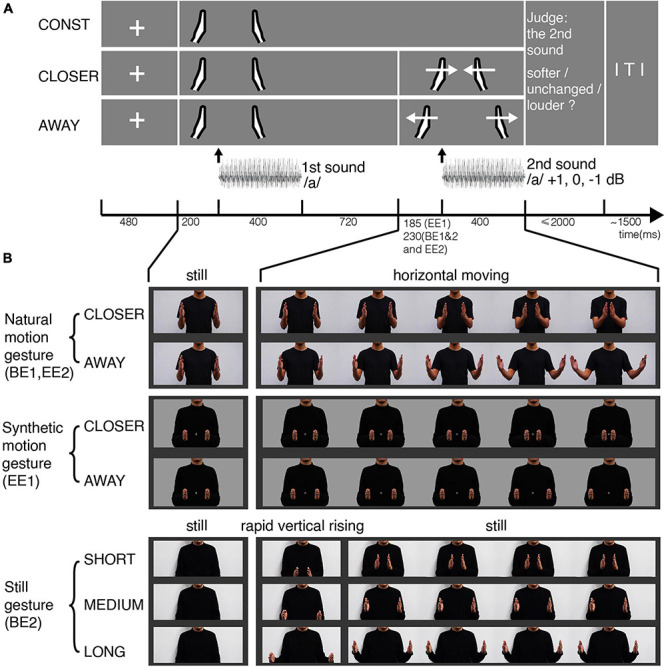
Experimental procedures and stimuli for the four experiments. **(A)** Experimental procedures in BE1, EE1, and EE2 (not in BE2). Each row depicts one condition in which the gestures vary. Participants first saw the still image of two hands in the middle position relative to the body and heard the vowel/a/. Then they either saw the motional gestures (CLOSER, AWAY) or the same still image (CONST). Meanwhile, they heard the same vowel/a/that either remained unchanged or increased 1 dB or decreased 1 dB from the first-time presentation. Participants performed a loudness judgment task. **(B)** Frames of gesture video clips. A male torso was shown from waist to chin in the middle of the screen. The hands always started from the middle position as a still image, followed by moving horizontally either toward each other (CLOSER) or away (AWAY). In BE1 and EE2, we used gestures with natural motion (the first row), recorded using a video camera and edited in Adobe Premiere. In EE1, we used gestures with computer-synthetic motion (the second row), controlled by a set of displacement functions (three periods: constant acceleration, uniform motion, constant deceleration). In BE2, we used still images of gestures with three different distances (the last row). The gestures appeared from the initial image of the body with a rapid vertical rising in two video frames and remained still until the end of the second sound.

In BE1, we used natural motional gestures in 1920 × 1080-pixel movie clips with a frame rate of 25 fps. The movie clips were made by combining video recordings of natural gestures and an audio recording of syllable/a/in a male voice. The gestures were performed by a male in front of his torso in black clothes, with gray backgrounds (Figure 1B, the first row). At the beginning of the videos, two hands appeared apart at an intermediate distance approximately the same width as the shoulder. The still frame was presented for 1320 ms, followed by videos of three different conditions. The hands keep constant (CONST condition, with no movement) for the rest of the trial, or they moved horizontally toward each other (CLOSER condition) or away from each other (AWAY condition) for 600 ms. The motion was naturally smooth, and the moving distances in CLOSER and AWAY were the same.

The auditory stimuli were a 400-ms vowel/a/adjusted in different levels of intensity, delivered through Sennheiser HD 280 headphones. The sound was extracted from a recording (sampling rate 44.1 kHz) of protracted pronunciation with steady intensity and then ramped in the first and last 20-ms duration. We only used one auditory token/a/because different tokens were hard to equalize to have the same loudness. Tokens with different perceived loudness would add confounding effects to the loudness-change judgment. We measured the sound intensity by using a sound meter (AWA5636, Aihua) with an acoustic coupler (ear simulator, AWA6160, Aihong) for circumaural headphones. We then calibrated the output sound intensity levels (see description below) by adjusting the sound wave files.

As shown in Figure 1A, at the beginning of each trial, a fixation cross was presented on the center of the screen for 480 ms, followed by the still image of two hands with an intermediate distance presented for 1320 ms. The first sound was played 200 ms after the onset of the still hands. The duration of the sound was 400 ms. Seven hundred twenty milliseconds after the sound offset, a video of gesture motion was presented in the CLOSER and AWAY conditions. In the CONST condition, the hands remained still. The stimulus onset asynchrony (SOA) between the onset of the gesture motion and the onset of the target sound (the second sound) was set at 230 ms in BE1. This sound latency was selected for three reasons. First, gestures usually precede articulations in natural conversations (Butterworth and Beattie, 1978; Morrel-Samuels and Krauss, 1992). Second, this amount of interval would be enough for the gesture to predictively modulate the auditory processing while still fall in the effective integration time window (van Wassenhove et al., 2007). Third, this interval would allow visual information to pre-modulate the auditory cortex or polymodal areas (Besle et al., 2008; Schroeder et al., 2008; Arnal et al., 2009; Talsma, 2015).

The intensity of the first sound was randomly selected from 55, 60, and 65 dB, while the intensity change of the second sound was randomly selected from −1, 0, and +1 dB relative to the first sound in that trial. We used the 1-dB step because the effect size of gestures’ cross-modulation could be small. Intensity change of 1 dB is around JND of most people with normal hearing (Johnson et al., 1993). The participants were given a maximum of 2 s to judge whether the second sound was softer, the same, or louder than the first sound. The inter-trial intervals (ITIs) were 1500 ms with no jitter because the study focuses on the interaction between the gestures and perception of the second sound. Three gestures and three intensity changes in the second sound were fully crossed and yielded nine conditions. In this experiment, 648 trials with 72 trials for each condition were divided into six blocks. All conditions were evenly distributed across blocks and were randomly presented in each block. The experiment was programmed and presented by using a Python package, Psychopy.

#### Behavioral Experiment 2 (BE2): The Effects of Still Images of Gestures on the Judgment of Loudness Changes

In BE2, we replaced motional gestures with still images of gestures (Figure 1B, the last row). The initial image was the torso, followed by four different conditions. The hands may not appear (NO-GESTURE), or they appeared at different distances apart – SHORT (the final frame of the CLOSER clip), MEDIUM (same as CONST), and LONG (the final frame of the AWAY clip). We added two frames of transitional motion to make them appear naturally. The two hands quickly moved up vertically from outside the bottom edge of the frame to the height of the chest. The hands then remained still until the end of the second sound.

We used in BE2 similar trial procedures as in BE1. Three still gestures (SHORT, MEDIUM, and LONG) appeared at the same time point when the gestures in BE1 started to move. No gestures appeared in the control condition (NO-GESTURE). Four types of visual displays and three sound intensity changes yielded 12 conditions in BE2.

#### EEG Experiment 1 (EE1): Comparing the Early Auditory ERP Responses Between Conditions of Different Gestures

In EE1, to make sure that the two motional gestures would elicit a similar response, we synthesized the movement of gestures in Python (Figure 1B, the second row). First, we limited the movement ranges of the two hands within the torso boundary to avoid a sudden contrast change. Second, we used a set of displacement functions with three stages (constant acceleration, uniform motion, and constant deceleration) to make the synthesized motion as natural as possible. Thirdly, we increased the video frame rate to 80 fps for smoother motion. We also shrank the clip frame to 613 × 318 pixels. As a result, the torso was within a visual range of 3.4°, both lateral and vertical, from fixation. The maximum horizontal range of the gesture movements was 3.1° lateral from fixation.

The trial procedure of EE1 was the same as in BE1 (Figure 1A). More control conditions were included in EE1 to quantify the neural responses of modulation effects. Specifically, a total of 14 conditions (Supplementary Table 1) were divided into three categories: audiovisual (AV), auditory-only (A), and visual-only (V). In the AV category, six conditions were included: two motional gestures (CLOSER and AWAY) were fully crossed with three intensity changes (−1, 0, +1 dB relative to the first sound). In the A category, another three conditions were included: one still gesture (CONST) and one fixation-only (BLANK) were fully crossed with three intensity changes (−1, 0, +1 dB). In the V category, additional two conditions were included: the two motional gestures (CLOSER and AWAY) were presented without any sound. We included 48 trials for each condition in the AV and A categories and 72 trials for each condition in the V category. A total of 576 trials in AV and A conditions were mixed together and evenly divided into 12 sets. A total of 144 trials in V conditions were also divided into 12 sets. The whole experiment contained 12 blocks. Each block included two parts – the first part contained a mix of AV and A conditions, and the second part contained only V conditions. The stimuli in each part were randomly presented. The same gesture appeared in no more than two consecutive trials. After the main experiment, participants went through an intensity localizer block, in which they passively listened to a sequence of 140 1-kHz pure tones at an average interstimulus interval (ISI) of 1 s, jittered between 800 and 1200 ms. Tones with two levels of intensity (67 and 69 dB) were randomly presented, with each intensity level presented 70 times. The intensity localizer was aimed to check if the sound intensity level *per se* would induce different ERPs.

Visual stimuli were presented *via* a display screen of Dell S2417DG with a resolution of 1920 × 1080 and a refresh rate of 165 Hz. The graphic card was GeForce RTX 2060. We fixed the intensity of the first sound at 70 dB to simplify EEG experiments and increase power. The intensity change of the second sound was randomly selected from −1, 0, and +1 dB relative to the first sound in that trial. Sounds were delivered through plastic air tubes connected to foam earpieces (ER-3C Insert Earphones; Etymotic Research). The sound intensities were measured using a sound-level meter (AWA5636, Aihua) with the acoustic coupler for insert earphones (occluded ear simulator, AWA6162, Aihong). Further, we adjusted the SOA between the onset of gesture motion and the onset of the second sound to 185 ms because the audiovisual integration has a high probability of occurring in a time window of 0–200 ms and is likely skewed toward the later part of this window (van Wassenhove et al., 2007). The pure tones (sampling rate of 44.1 kHz; duration of 400 ms) in the intensity localizer were generated in Praat (Boersma and Weenink, 2021).

To control the timing of visual and auditory stimuli precisely, we recorded the onset timing of both the visual and auditory stimuli *via* StimTracker Duo (Cedrus) system (the trigger box). A light sensor was attached to the lower-left corner of the monitor and connected to the trigger box. The acoustic signals were split into the trigger box (another went to the earphones). The onset time of each physical stimulus was captured with a sampling frequency of 1 kHz, which provided a set of temporal markers to the physical stimuli measured in the timeline of EEG recordings. The actually measured distribution of SOAs in EE1 had a mean of 185.5 ms and an SD of 10.4 ms. We did not align the stimuli to the refresh rate of the screen. However, the refresh rate of the screen was more than double the corresponding video frame rate in both EEG experiments.

#### EEG Experiment 2 (EE2): Comparing the Early Auditory ERP Response Between Trials of Different Loudness Judgment to the Same Sound

In EE2, we examined the modulation effects of gestures by quantifying the neural responses in trials with different perceptual judgments to the same stimuli. We used the same gestures with natural motion (Figure 1B, the first row) as in BE1 because there was no need to control the visual responses to CLOSER and AWAY gestures in this experiment. We only compared between conditions within either gesture. Moreover, we used the same SOA (230 ms) between the onset of motional gesture and the onset of the second sound as in BE1. The reason to increase the SOA was to further separate the ERP responses to the second auditory stimulus from those driven by the motion gestures so that our questions can be better addressed. The neural responses take time to accumulate so that EEG signals can be recorded. For example, the early perceptual components in visual and auditory domains can take about 200 ms – the classic N1/P2 components. The actually measured distribution of SOAs in EE2 had a mean of 229.9 ms and an SD of 8.9 ms. Similar trial procedures as in BE1 were used with several modifications to yield enough trials of biased responses to the same auditory stimuli. First, we excluded the CONST conditions. Second, we fixed the intensity of the first sound at 68 dB. Third, we adjusted the proportions of intensity changes (−1, 0, and +1 dB) to a ratio of 1:5:1. Reasonable percentages of −1 and +1 dB intensity changes were included to convince the participants that the intensity did vary and to avoid any strategies. A large portion of trials was intensity unchanged (0 dB) so that enough trials would be obtained in situations of different loudness judgment to the same intensity. In total, six conditions were included in EE2 (2 motional gestures × 3 levels of intensity change). A total of 672 trials were divided into 12 blocks. The presentation order was randomized in each block. EE2 was carried out using a display screen of Dell E2214Hv with a resolution of 1920 × 1080 and a refresh rate of 60 Hz. The graphic card was AMD Radeon HD 5450. The video stimuli were presented with 25 fps.

### Procedure

#### General Experimental Procedures for BE1, BE2, EE1, and EE2

BE1 and BE2 were carried out in a small room with participants sitting on a comfortable chair. Before each experiment, participants were given instructions on how to attend to the stimuli properly. They were asked to watch the hands, to pay attention to the sounds, and to make judgments based on the auditory stimuli. Importantly, they were explicitly told that the gestures and sound intensity changes were randomly paired. Participants went through a brief training to familiarize the changes in sound intensity. During training, they judged the loudness change of the second sound without the presence of the visual stimulus and with real-time feedback. After they passed the training, they went through a practice block to familiarize themselves with all the stimuli and tasks. We verbally confirmed that they could see the gestural motion easily and that they could hear the intensity changes in the practice block. During the experiments, participants were required to take a break for at least 1 min between two blocks.

EE1 and EE2 share the same procedure with BE1 and BE2 except for a few aspects. The two EEG experiments were carried out in an electromagnetically shielded and soundproof booth. The location of the chair was fixed to control the retinal angle of the visual stimuli. We asked the participants to fixate at the tiny cross displayed at the center between two hands, sit still, and avoid unnecessary head movement and eye blink during the trial.

#### EEG Data Acquisition

EEG signals were recorded with a 32-electrode active electrodes system (actiChamp system, Brain Products GmbH, Germany). Electrodes were placed on EasyCap, on which electrode holders were arranged according to the 10–20 international electrode system. Two additional electrooculogram (EOG) electrodes were used to monitor horizontal and vertical ocular movements, respectively. The ground electrode was placed at the forehead. Electrode impedances were kept below 10 kΩ. The data were continuously recorded in single DC mode, sampled at 1000 Hz and referenced online to the electrode Cz. The EEG data were acquired with Brain Vision PyCoder software and filtered online by the acquisition system using a low-pass filter (second order Butterworth) with a cutoff frequency of 200 Hz. A 50-Hz notch filter was applied to filter out AC noise online during EEG recordings. EEG data processing and analysis were conducted with customized Python codes, MNE-python (Gramfort et al., 2014), EasyEEG (Yang et al., 2018).

### Data Analysis

#### Behavioral Data Analysis of BE1, BE2, EE1, and EE2

For the behavioral data in each experiment, we calculated a judgment score in each condition to characterize a participant’s judgment preference. The score was obtained by averaging all the judgments (1 for choosing louder, 0 for unchanged, and −1 for softer) across trials. We also calculated in each condition the participants’ accuracy – the ratio of the number of correct trials to the total number of trials. We applied repeated measure two-way analyses of variance (ANOVA) to the judgment scores and accuracy, respectively, with the factors of intensity change and gesture, followed by *post hoc* pairwise comparisons using *t*-tests with Bonferroni correction. We checked the normality of ANOVA residuals by visual inspection of the Q–Q plot and Shapiro–Wilk test. The residuals were approximately normal. We checked the sphericity assumption using Mauchly’s Test of Sphericity. We applied the Greenhouse–Geisser correction when the sphericity assumption was violated. In EE1, we also calculated the accuracy of behavioral judgment for the BLANK condition in each intensity change. The accuracy without any gestural influence in the training session of each experiment and in the EE1 BLANK condition was compared with the 0.33 chance level by using a one-sample one-tailed *t*-test.

Furthermore, we calculated the bias ratios in each of the three intensity changes to index how the manual gestures influence the loudness judgment to different intensity changes. It is a summary statistic based on the confusion matrix (the percentage of choice responses with respect to the total trial in that condition) shown in Supplementary Table 2. For ±1 dB intensity change, there were two kinds of judgment biases. The response could be off the actual intensity change by 1 level (level-1 bias), such as responding “unchanged” when the second sound increased or decreased by 1 dB. The bias could also be off by 2 levels (level 2 bias), such as responding “louder” when the intensity change was −1 dB and vice versa. For 0 dB (no intensity change), only level-1 bias could be induced. For −1 and 0 dB intensity change, the judgment bias of louder percepts was obtained for each gesture: bias ratio = frequency of louder bias/frequency of all bias. For +1 dB intensity change, the judgment bias of softer percept was calculated for each gesture: bias ratio = frequency of softer bias/frequency of all bias. We applied planned paired *t*-tests to the bias ratios between different gestures in each level of intensity change.

#### EEG Data Analysis

For each participant, data were band-pass filtered (0.1–30 Hz, Kaiser windowed FIR filter) offline and re-referenced to the average potential of all the EEG electrodes. For EE1, epochs were extracted according to the conditions (AV conditions, −310 to 400 ms, time-locked to the onset of the second sound; A conditions, −100 to 400 ms, time-locked to the onset of the second sound; V conditions, −100 to 600 ms, time-locked to the onset of the motional gesture; intensity localizer, −100 to 400 ms, time-locked to the onset of the tones). All epochs were baseline-corrected using the 100-ms pre-stimulus data, except for the AV conditions, which were baseline-corrected using the 100-ms pre-motion data (−310 to −210 ms). For EE2, epochs were extracted from −350 to 400 ms time-locked to the onset of the second sound. All epochs were baseline-corrected using the pre-motion data from −350 to −250 ms.

To ensure data quality, epochs with peak-to-peak amplitude exceeding 100 μV were automatically excluded, and epochs with artifacts that resulted from eye blinks and other muscle movements were manually rejected. We identified eye blinks by visual inspection of the two EOG channels. We identified and removed muscle artifacts also by visual inspection. The remaining epochs were used to obtain the ERP in each condition. An average of 30.2 trials (SD = 7.2, out of 48 trials per condition) of AV conditions and an average of 45.9 trials (SD = 10.7, out of 72 trials per condition) of V conditions were included in EE1. An average of 52.6 trials (SD = 19.3) were included in EE2 (the total number of trials in each condition varied depending on loudness judgment). Two participants were excluded from EE1, and three participants were excluded from EE2 because they either produced many artifacts (more than 50%) or made almost identical behavior responses in all conditions (probably not following instructions nor paying attention to the task). Their behavioral data were also excluded from the analysis.

For EE1, the EEG epochs were averaged and created an ERP response in each condition. Instead of selecting sensors, we calculated a more conservative index, the global field power (Murray et al., 2008). GFP, calculated as the root mean square of data in all sensors, represents the amount of energy change in all sensors throughout the time. GFP provides more holistic and unbiased information (Murray et al., 2008). We applied a temporal cluster analysis (Maris and Oostenveld, 2007) to compare the GFP waveforms of two conditions. Specifically, we first calculated a paired *t*-statistics between the two conditions at each time point. Then, temporal clusters were formed with more than two adjacent time points where the corresponding *p*-value was above the threshold (0.05). We summed all the *t*-values within each temporal cluster as its summary empirical statistics. To form a distribution of the null hypothesis, we permutated the condition labels 10,000 times and collected the maximum cluster sum-*t* value in each permutation. Finally, the summary empirical statistics of each temporal cluster identified in the original data were tested in the permutation distribution of max-*t* values. The same temporal cluster analysis was separately applied in the AV conditions, V conditions, A conditions, and the intensity localizers in the absence of visual modulation.

For EE2, to examine how neural responses were modulated as a function of perception to the same auditory stimuli, only the data in the conditions of 0 dB (no intensity change) were used in EEG analysis. Data were divided into three groups based on participants’ judgment in either gesture level (CLOSER and AWAY). The three groups were (1) trials of “softer” perceptive shift (choosing softer), (2) trials of “louder” perceptive shift (choosing louder), and (3) trials of no perceptive shift (choosing unchanged). We applied the ERP component analysis and temporal cluster analysis to the comparison between “softer” perceptive shift and no perceptive shift in the CLOSER condition, as well as to the comparison between “louder” perceptive shift and no perceptive shift in the AWAY condition. The exact N100 peak latency varied in individual participants. Therefore, in the ERP component analysis, the N100 was automatically located with an in-house algorithm (Wang et al., 2019) for each participant in a pre-determined time range (65–135 ms). We took an average of the amplitudes in a 20-ms window centered at the individual N100 peak as the N100 response magnitude. For all paired comparisons, the numbers of epochs in the pair of conditions were equalized with the function of “equalize_epoch_counts” included in the MNE toolbox. Basically, this function equalizes the number of trials in two conditions by selecting trials in the conditions that have more trials. The criterion of selection is that the selected trials would occur as close as possible in time to the trials in the other condition.

## Results

The analysis of the training data (Supplementary Figure 1) showed that participants were able to discriminate the intensity changes above the chance level (0.33) without gestural influence [BE1, *M* = 0.47, SD = 0.12, *t*(14) = 4.34, *p* < 0.001, *d*_*z*_ = 1.09; BE2, *M* = 0.41, SD = 0.09, *t*(11) = 2.97, *p* = 0.006, *d*_*z*_ = 0.86; EE1, *M* = 0.47, SD = 0.14, *t*(20) = 4.21, *p* < 0.001, *d*_*z*_ = 0.92; EE2, *M* = 0.43, SD = 0.09, *t*(16) = 3.95, *p* < 0.001, *d*_*z*_ = 0.96].

### Behavioral Experiment 1 (BE1): The Effects of Motional Gestures on the Judgment of Loudness Changes

**FIGURE 2:**
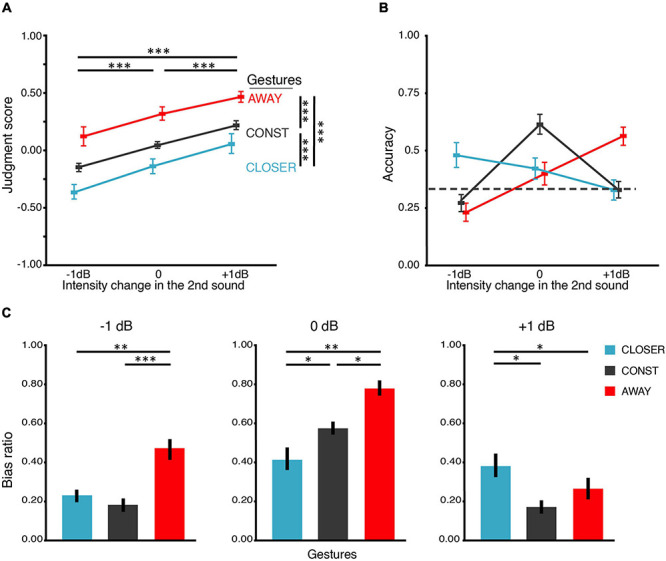
Results of BE1. **(A)** Judgment score. BE1 investigated how motional gestures influenced loudness perception. The judgment score was obtained by averaging all the responses in which “1” for “louder,” “0” for unchanged, and “–1” for softer. Therefore, the judgment score reflects the overall judgment tendency, where 0 stands for no change, positive for louder and negative for softer. Participants can correctly identify the intensity changes – the judgment scores increased as the intensity increased in all gesture conditions. Moreover, gesture modulated the loudness judgment – in all levels of intensity change. Judgment scores in the AWAY gesture condition were larger than those in CONST, and judgment scores in CONST were larger than those in CLOSER. **(B)** Accuracy of the behavioral judgments about intensity change. We obtained accuracy by calculating the portion of trials that participants correctly identified the intensity change. There is an interaction between the factors of gesture and intensity change. The interaction was driven by higher accuracies in conditions where the changes in intensity and gestures were consistent. The dashed line indicates the chance level (0.33). **(C)** Bias ratios. The bias ratio was calculated to index how the manual gestures influence the loudness judgment to different intensity changes. For –1 dB intensity change (left panel) and 0 dB intensity change (middle panel), the judgment bias of louder percepts was obtained for each gesture: bias ratio = frequency of louder bias/frequency of all bias. For +1 dB intensity change (right panel), the judgment bias of softer percept was calculated for each gesture: bias ratio = frequency of softer bias/frequency of all bias. The results indicate that the judgment was biased toward the “matched” gesture in all intensity changes. All error bars indicate ±one SEM. ^∗^*p* < 0.05; ^∗∗^*p* < 0.01; ^∗∗∗^*p* < 0.001.

Response accuracy was differentially influenced by gestures across intensity changes (Figure 2B). The average accuracy was around 0.5 where the gesture direction and intensity change matched, and the accuracy was lower than that when the gesture direction and intensity change did not match. ANOVA revealed that the main effect of intensity change on accuracy was not significant [*F*(1.16,28) = 3.98, *p* = 0.058, η*_*p*_*^2^ = 0.22, ε = 0.58], and the main effect of gestures was not significant [*F*(2,28) = 0.64, *p* = 0.535, η*_*p*_*^2^ = 0.04, ε = 0.76] either. Crucially, there was an interaction between gesture and intensity change [*F*(1.48,56) = 14.62, *p* < 0.001, η*_*p*_*^2^ = 0.41, ε = 0.31]. Pairwise *t*-tests revealed that the accuracies of CONST (*M* = 0.27, SD = 0.14) and AWAY (*M* = 0.23, SD = 0.15) were lower than the accuracy of CLOSER (*M* = 0.47, SD = 0.20) in −1 dB intensity change [*t*(14) = 4.20, *p* < 0.01, *d*_*z*_ = 1.21; *t*(14) = 3.72, *p* = 0.02, *d*_*z*_ = 1.40]. The accuracies of CLOSER (*M* = 0.42, SD = 0.18) and AWAY (*M* = 0.40, SD = 0.19) were lower than the accuracy of CONST (*M* = 0.61, SD = 0.17) in 0 dB intensity change [*t*(14) = 4.51, *p* = 0.004, *d*_*z*_ = 1.11; *t*(14) = 4.05, *p* = 0.01, *d*_*z*_ = 1.20]. In addition, the accuracies of CLOSER (*M* = 0.33, SD = 0.17) and CONST (*M* = 0.33, SD = 0.14) were lower than the accuracy of AWAY (*M* = 0.56, SD = 0.16) in +1 dB intensity change [*t*(14) = 3.54, *p* = 0.03, *d*_*z*_ = 1.46; *t*(14) = 4.05, *p* = 0.01, *d*_*z*_ = 1.57]. That is, the highest accuracy was found where the gestural direction matched with the intensity change (CLOSER with intensity −1 dB, AWAY with intensity +1 dB, CONST with intensity unchanged). On the contrary, accuracy was much lower where the gestural direction and the intensity change did not match.

Both gesture and intensity change positively affected the judgment scores (Figure 2A). On average, the judgment score monotonically increased as a function of the intensity change (−1, 0, +1 dB) or the gesture (CLOSER, CONST, AWAY). ANOVA showed that the main effect of intensity change was significant [*F*(1.16,28) = 50.61, *p* < 0.0001, η*_*p*_*^2^ = 0.78, ε = 0.58]. More importantly, the main effect of gesture was also significant [*F*(1.09,28) = 13.92, *p* = 0.002, η*_*p*_*^2^ = 0.450, ε = 0.54]. However, the interaction was not significant [*F*(4,56) = 1.19, *p* = 0.30, η*_*p*_*^2^ = 0.08, ε = 0.19]. The pairwise *t*-tests revealed that the judgment scores under AWAY (*M* = 0.31, SD = 0.23) were higher than the judgment scores under CONST (*M* = 0.04, SD = 0.08) [*t*(44) = 6.41, *p* < 0.0001, *d*_*z*_ = 1.13]. The judgment scores under CONST (*M* = 0.04, SD = 0.08) was higher than the judgment scores under CLOSER (*M* = –0.15, SD = 0.26) [*t*(44) = 5.46, *p* < 0.0001, *d*_*z*_ = 0.72]. These results suggest that: (1) participants were able to detect intensity changes (not by pure guessing); (2) The moving directions of the gestures were in line with the bias they caused in intensity judgment.

The bias ratio characterized the direction and extent to which gestures biased the judgments (Figure 2C). When the intensity change was −1 dB, AWAY (*M* = 0.47, SD = 0.21) induced higher ratio of level-2 bias (judge louder) than CONST (*M* = 0.18, SD = 0.13) [*t*(14) = 4.94, *p* < 0.001, *d*_*z*_ = 1.67] and CLOSER (*M* = 0.23, SD = 0.13) [*t*(14) = 3.83, *p* = 0.006, *d*_*z*_ = 1.38]. When the intensity change was +1 dB, CLOSER (*M* = 0.38, SD = 0.22) also induced higher ratio of level-2 bias (judge softer) than CONST (*M* = 0.17, SD = 0.12) [*t*(14) = 4.71, *p* = 0.001, *d*_*z*_ = 1.22] and AWAY (*M* = 0.26, SD = 0.21) [*t*(14) = 2.94, *p* = 0.03, *d*_*z*_ = 0.56]. When the intensity change was 0 dB, AWAY (*M* = 0.78, SD = 0.16) produced higher ratio of louder bias than CONST (*M* = 0.57, SD = 0.12) [*t*(14) = 4.55, *p* ≤ 0.001, *d*_*z*_ = 1.49] and CONST produced higher ratio of louder bias than CLOSER (*M* = 0.41, SD = 0.22) [*t*(14) = 3.25, *p* = 0.097]. The analyses using bias ratios further suggested that the gestures biased the judgments of loudness changes when they were inconsistent. BE1 results provided overall behavioral evidence supporting the hypothesis that gestures influence loudness perception.

### Behavioral Experiment 2 (BE2): Still Gesture Images Modulated Judgment of Loudness Less Than Motional Gestures

**FIGURE 3:**
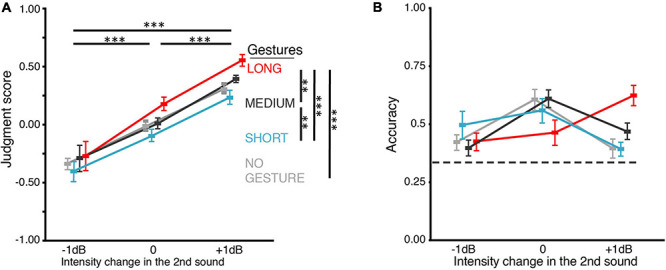
Results of BE2. **(A)** Judgment score. BE2 investigated how the gestures in still images influenced loudness perception. Because for each of the three gestures, no gesture was shown before the hands showed up, we included a NO-GESTURE as the baseline condition. The loudness judgment was positively influenced by both the intensity change and still gesture. However, the difference was neither significant between MEDIUM and NO-GESTURE nor between SHORT and NO-GESTURE. The influence of still gestures on loudness judgment was smaller than motional gestures in BE1 (Figure 2A). In addition, the judgment scores were not different between any pair of gestures in –1-dB intensity change. **(B)** Accuracy of the behavioral judgments about intensity change. When intensity changes were –1 or 0 dB, no gesture differed in its influence on judgment accuracy, though the overall interaction between the factors of gesture and intensity change was significant. The dashed line indicates the chance level (0.33). All error bars indicate ±one SEM. *p < 0.05; ^∗∗^*p* < 0.01; ^∗∗∗^*p* < 0.001.

The goal of BE2 was to examine whether the influence of gestures on loudness judgment was only due to the distance between hands in the gestures. Therefore, in BE2, we replaced motional gestures used in BE1 with still images of gestures. The judgment score increases as the intensity change goes from −1 dB to 0 and to +1 dB but is only moderately influenced by gestures (Figure 3A). The statistical results suggest that both gesture and intensity change positively affected the judgment score. ANOVA showed that the main effects of both intensity change and gesture were significant [*F*(1.28,22) = 114.20, *p* < 0.0001, η*_*p*_*^2^ = 0.91, ε = 0.64; *F*(1.34,33) = 8.35, *p* = 0.007, η*_*p*_*^2^ = 0.43, ε = 0.45]. The interaction was not significant [*F*(6,66) = 3.34, *p* = 0.071, η*_*p*_*^2^ = 0.23, ε = 0.26]. For the judgment scores, pairwise *t*-tests (Bonferroni corrected) showed that LONG (*M* = 0.15, SD = 0.15) was higher than MEDIUM (*M* = 0.04, SD = 0.12) [*t*(35) = 3.65, *p* < 0.005, *d*_*z*_ = 0.33]; MEDIUM was higher than SHORT (*M* = −0.09, SD = 0.20) [*t*(35) = 3.99, *p* = 0.002, *d*_*z*_ = 0.38]; LONG was higher than NO-GESTURE (*M* = −0.01, SD = 0.10) [*t*(35) = 5.61, *p* < 0.0001, *d*_*z*_ = 0.48]. However, the judgment scores of SHORT and MEDIUM were not significantly different from NO-GESTURE [*t*(35) = 2.20, *p* = 0.21, *d*_*z*_ = 0.23; *t*(35) = 2.67, *p* = 0.07, *d*_*z*_ = 0.17]. Moreover, the judgment of loudness change was not modulated by the gestures in −1-dB intensity change: the judgment scores were not different between any pair of gestures. Finally, the differences of judgment scores across the three still gestural conditions (SHORT, MEDIUM, LONG) in BE2 (Figure 3A) were less than the differences of judgment scores across the three motion gestural conditions (CLOSER, CONST, AWAY) in BE1 (Figure 2A).

Response accuracy further showed a difference in BE2, as compared to that in BE1. In BE2 (Figure 3B), although ANOVA still showed that gesture interacted with intensity change [*F*(2.69,66) = 9.05, *p* < 0.0001, η*_*p*_*^2^ = 0.45, ε = 0.30], the pairwise *t*-tests failed to show any difference between gestures in −1 and 0 dB intensity changes. When the intensity change was +1 dB, only LONG (*M* = 0.62, SD = 0.15) showed higher accuracy than NO-GESTURE (*M* = 0.39, SD = 0.15) [*t*(11) = 7.9, *p* = 0.0001, *d*_*z*_ = 1.5] and SHORT (*M* = 0.39, SD = 0.10) [*t*(11) = 5.47, *p* = 0.004, *d*_*z*_ = 1.82]. These results suggested that the visual-motor information in the motional gestures contributed more greatly to the modulation effects on loudness judgment than the final distance between two hands. We used motional gestures to investigate further the dynamics of the modulation effects in the following EEG experiments.

### EEG Experiment 1 (EE1): Gestures Modulated Early Neural Responses in Loudness Perception

#### Behavioral Results

**FIGURE 4:**
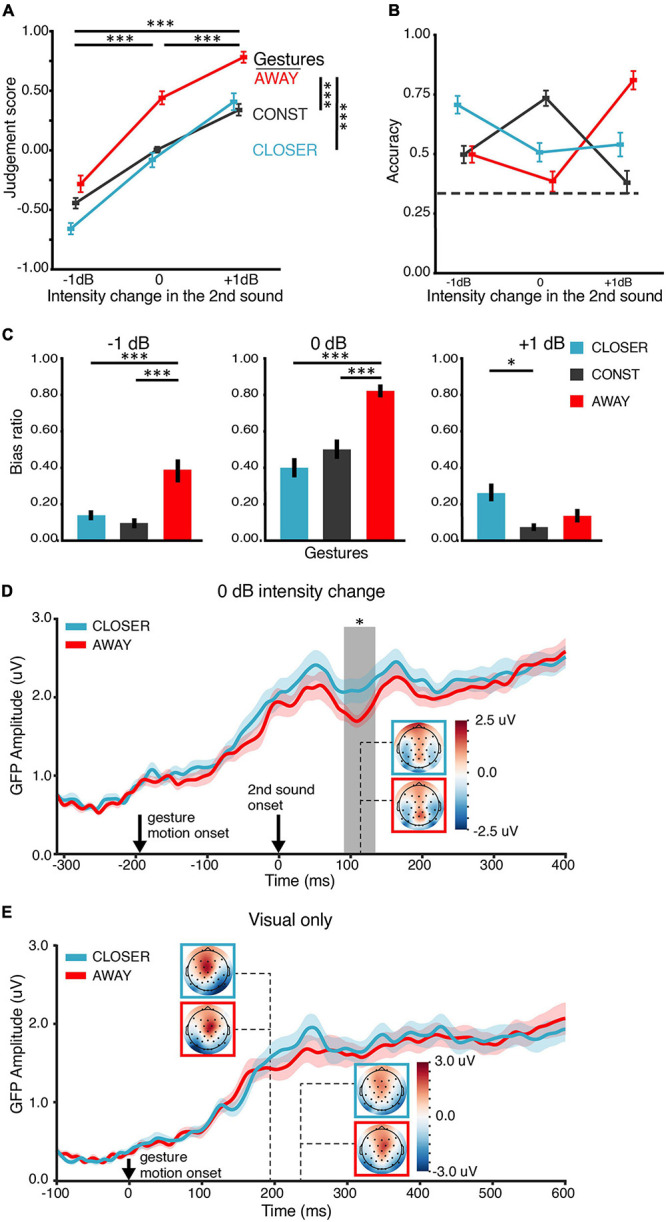
Results of EE1. **(A)** Judgment score. Behaviorally, both gesture and intensity change positively affected the judgment of loudness changes. The main effects were similar to the results in BE1 in Figure 2A. **(B)** Accuracy of the behavioral judgment about intensity change. An interaction between the factors of gesture and intensity change was observed, consistent with the main results in BE1 in Figure 2B. The dashed line indicates the chance level (0.33). **(C)** Bias ratios. The judgment was biased toward the “matched” gesture 0-dB-intensity-change conditions. **(D)** ERP responses to the second sound in the 0-dB-intensity-change conditions were modulated by the gestures. Gesture CLOSER elicited a stronger ERP response than gesture AWAY at around 110 ms after the onset of the second sound. Solid lines in each plot indicate the grand mean global field power (GFP) waveform. The shades around the lines represent +, – one SEM (*n* = 21). Response topographies are shown in colored boxes with dashed lines pointing to their latencies. The colored boxes use the same color schemes as the waveform responses to indicate different conditions. The gray vertical rectangular shade indicates the temporal cluster (91–135 ms) in which the two GFPs were significantly different in the temporal cluster analysis. **(E)** ERP responses to video stimuli in the visual-only (V) conditions. The visual ERP responses to gesture CLOSER and AWAY were not significantly different, suggesting that different visual stimuli evoked similar early visual responses and the observed effects in panel **(D)** were not caused by different visual gesture stimuli. The depicting formats are the same as in panel **(D)**. The error bars indicate ±one SEM. ^∗^*p* < 0.05; ^∗∗^*p* < 0.01; ^∗∗∗^*p* < 0.001.

The behavioral results in EE1 replicated those in BE1. In EE1, both gesture and intensity change positively affected the judgment scores (Figure 4A). ANOVA showed that the main effects of both intensity change and gesture were significant [*F*(1.23,40) = 139.55, *p* < 0.0001, η*_*p*_*^2^ = 88, ε = 0.62; *F*(2,40) = 44.26, *p* < 0.0001, η*_*p*_*^2^ = 0.69, ε = 0.83]. The interaction was also significant [*F*(4,80) = 12.82, *p* < 0.001, η*_*p*_*^2^ = 0.39, ε = 0.36]. Pairwise *t*-tests (Bonferroni corrected) showed that the judgment scores under AWAY (*M* = 0.30, SD = 0.18) was higher than the judgment scores under CONST (*M* = −0.04, SD = 0.11) [*t*(62) = 9.88, *p* < 0.0001, *d*_*z*_ = 0.76] and CONST was higher than CLOSER (*M* = −0.12, SD = 0.18) [*t*(62) = 2.53, *p* = 0.04, *d*_*z*_ = 0.18]. Second, gesture interacted with intensity change in terms of their effects on accuracy [*F*(4,84) = 54.94, *p* < 0.0001, η*_*p*_*^2^ = 0.74, ε = 0.43] (Figure 4B).

The response accuracy for the BLANK condition (where only the fixation was shown, Supplementary Figure 2) was above the 0.33 chance level in each intensity change [−1 dB, *M* = 0.51, SD = 0.16, *t*(20) = 5.14, *p* < 0.0001, *d*_*z*_ = 1.12; 0 dB, *M* = 0.69, SD = 0.14, *t*(20) = 11.97, *p* < 0.0001, *d*_*z*_ = 2.61; +1 dB, *M* = 0.45, SD = 0.19, *t*(20) = 2.86, *p* = 0.005, *d*_*z*_ = 0.62]. These results confirmed that participants were able to discriminate all three intensity changes when no gesture was shown.

The pattern of the bias ratio (Figure 4C) in EE1 was similar to that in BE1. The judgment was biased toward the “matched” gesture in all intensity changes. CLOSER and AWAY biased the choice toward opposite directions in 0-dB intensity change: the louder bias ratio of AWAY (*M* = −0.83, SD = 0.13) was larger than the louder bias ratio of CLOSER (*M* = 0.40, SD = 0.22) [*t*(20) = 8.97, *p* < 0.0001, *d*_*z*_ = 2.40]. These results suggest that (1) participants were able to detect the real changes of intensity (not by pure guessing); (2) The influence of gestures on the judgments of loudness change positively correlated to the direction of movement in gestures, and (3) The gestures biased the judgments of loudness changes when the direction of change was not congruent across modalities.

#### EEG Results

If the gestures CLOSER and AWAY modulated loudness perception rather than decisional processes, the modulation effects should be observed in ERPs at early latencies (e.g., ∼100 ms) rather than at late latencies. For the 0 dB intensity change, the GFP waveforms in both gesture conditions rose following the gesture motion onset and increased again about 50 ms after the second sound’s onset (Figure 4D). An apparent diverge of the two waveforms was observed around 100-ms latency. CLOSER evoked a larger response than AWAY in a temporal cluster from 91 to 135 ms (*p* = 0.039). The effects of gestures on early auditory responses were not caused by visual responses because the two gestures elicited similar GFPs across the time in visual-only (V) conditions (Figure 4E). No significant cluster was found in the temporal cluster analysis. For the −1 and +1-dB intensity change, respectively, no significant difference was found in the GFP waveforms of the two gesture conditions.

It was somewhat surprising that CLOSER evoked larger auditory responses than AWAY for the 0-dB intensity change. This modulation pattern could arise from the interaction between gestures and a particular neural response profile to physical stimuli in the current experimental setting. Therefore, we further investigated the neural response profile to auditory stimuli with different levels of intensity without motional gestures. First, we examined the ERP responses in auditory-only (A) conditions in which different intensity changes were presented with the still image of the CONST gesture. No difference was found in the temporal cluster analysis (Supplementary Figure 3A). Also, we did not find any difference between the low-intensity and high-intensity conditions in the intensity localizer (Supplementary Figure 3B). These results suggest that the ERP difference we found in the AV conditions was specific to gestural modulation.

In summary, the behavioral results in EE1 were similar to those in BE1 and supported that audiovisual gesture information biased the judgments of loudness changes. More importantly, the modulation effects were observed in auditory neural responses at an early latency (around 110 ms after the second sound onset). The modulation pattern in the 0-dB-intensity-change conditions was somewhat surprising and specific to the gestures. Therefore, to replicate the results of EE1 and to provide further evidence about across-modal effects on loudness perception, we carried out EE2 in which the modulation effects of gesture were examined as a function of loudness perception to the same physical stimuli.

### EEG Experiment 2 (EE2): Changes of Loudness Perception Were Reflected by the Modulation in Early Auditory Responses

#### Behavioral Results

**FIGURE 5:**
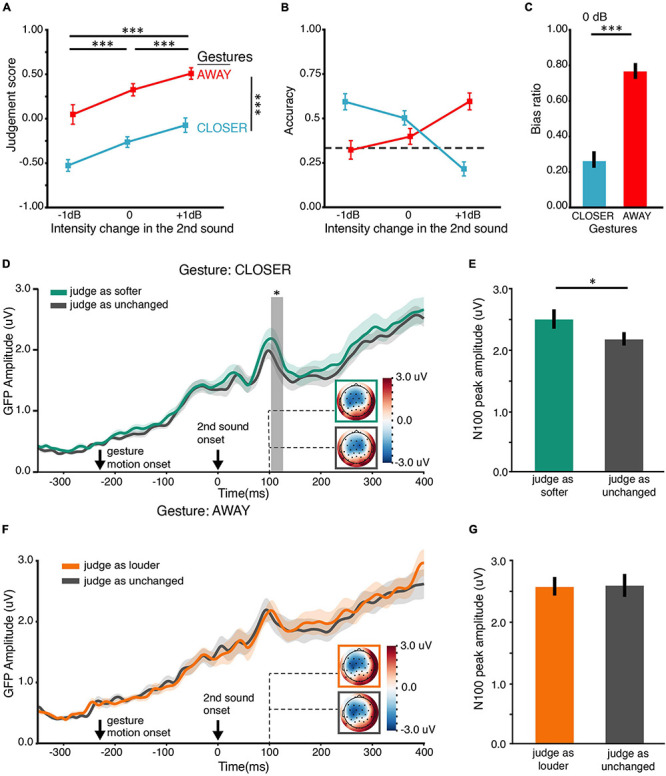
Results of EE2. **(A)** Judgment score. Behaviorally, both gesture and intensity change positively affect the judgment of loudness changes, like the results in BE1 in Figure 2A. **(B)** Accuracy of the behavioral judgment about intensity change. An interaction between the factors of gesture and intensity change was observed, consistent with the results in BE1 (Figure 2B). The interaction was driven by the boost of accuracy by the CLOSER gesture in –1-dB intensity change and by the AWAY gesture in the +1-dB intensity change. **(C)** Bias ratios in the 0 dB intensity change conditions. The bias ratio was calculated as the judgment biased toward a louder percept: bias ratio = frequency of louder bias/frequency of all bias. AWAY biased participants toward choosing louder (higher bias ratio) while CLOSER biased participants toward choosing softer (lower bias ratio); the actual intensity did not change. **(D)** ERP responses to the second sound with 0-dB intensity change in the CLOSER conditions as a function of loudness perception. Stronger ERP responses were observed at around 115-ms latency when the second sound was perceived as “softer” (green) than was perceived as unchanged (gray), although the stimuli were the same sound of 0 dB intensity change. The solid lines indicate the grand mean global field power (GFP) waveforms. The shades around the solid lines represent ±one SEM. Response topographies are shown in colored boxes with dashed lines pointing to their latencies. The colored boxes use the same color schemes as the waveform responses to indicate different conditions. The gray vertical shades indicate the temporal cluster (104–207 ms) in which the two GFPs were significantly different in the temporal cluster analysis. **(E)** Response magnitude of N100 component in “softer” perceptive shifts (green) and no perceptive shifts (gray), obtained by temporally averaging a 20-ms time window centered at the individual early peak latencies (100 ms) observed in panel **(D)**. The response magnitude of N100 was larger in “softer” perceptive shifts than that in no perceptive shifts. **(F)** ERP responses to the second sound of 0 dB intensity change in the AWAY conditions as a function of loudness perception. No difference between ERP responses was observed. The depicting formats are the same as in panel **(D)**. **(G)** Response magnitude of N100 components in “louder” perceptive shifts (orange) and no perceptive shifts (gray), obtained by temporally averaging a 20-ms time window centered at the individual early peak latencies (100 ms) observed in panel **(F)**. “Louder” perceptive shifts had a similar response magnitude to no perceptive shifts in the early auditory response of N100. ^∗^*p* < 0.05; ^∗∗^*p* < 0.01; ^∗∗∗^*p* < 0.001. Error bars indicate ±one SEM.

The behavioral response in BE1, EE1, and EE2 followed the same pattern (Supplementary Figure 4). The trends of the judgment score and accuracy in EE2 (Figures 5A,B) were similar to those in BE1. We applied the same statistical analyses used in BE1 to the behavioral data of EE2. The behavioral results were similar to those in BE1 and EE1, although we removed the still gesture CONST and increased the number of 0-dB-intensity-change trials. ANOVA showed that the main effects of both intensity change and gesture on the judgment scores were significant [*F*(1.04,32) = 21.82, *p* < 0.001, η*_*p*_*^2^ = 0.58, ε = 0.52; *F*(1,16) = 21.93, *p* < 0.001, η*_*p*_*^2^ = 0.58, ε = 1.00] (Figure 5A). However, the interaction was not significant [*F*(2,32) = 1.21, *p* = 0.31, η*_*p*_*^2^ = 0.07, ε = 0.98]. These results suggested that participants could detect the actual intensity change, and their judgments of loudness changes positively correlated with the direction of movement in gestures. Moreover, gesture interacted with intensity change in terms of their effects on accuracy [*F*(1.30,32) = 25.69, *p* < 0.0001, η*_*p*_*^2^ = 0.62, ε = 0.65] (Figure 5B). Especially, the louder bias ratio of CLOSER (*M* = 0.26, SD = 0.18) was significantly lower than the louder bias ratio of AWAY (*M* = 0.75, SD = 18) in 0 dB intensity change [*t*(16) = 3.88, *p* = 0.004, *d*_*z*_ = 0.61] (Figure 5C). These results indicated that when the second sound was identical to the first sound, gesture CLOSER drove participants toward “softer” bias, whereas gesture AWAY drove participants toward “louder” bias.

#### EEG Results

We designed the EE2 to further investigate the relation between neural modulations and loudness perception changes caused by gestures. Specifically, we examined how gestures changed the auditory neural responses as a function of subjective biases in loudness perception to the same physical stimuli. Based on what we found in EE1, we expected that “softer” bias (induced by gesture CLOSER) would have stronger ERP responses at early latency (N100) than no bias. Indeed, the ERP time course of “softer” perceptive shifts had larger responses than no perceptive shifts shortly after 100-ms latency (Figure 5D). A cluster from 104 to 127 ms (*p* = 0.047) was found by the temporal cluster analysis. This was consistent with results in the paired *t*-test on N100 component response magnitude [*t*(16) = 2.17, *p* = 0.045, *M*_diff_ = −0.34 μV, *d*_*z*_ = 0.53] (Figure 5E). Note that these two conditions (“softer” perceptive shifts and no perceptive shifts) were identical in all physical aspects. The only difference between them was in subjective judgment. These results suggested that the bias in loudness perception induced by gesture CLOSER was accompanied by an early perceptive modulation at around 100 ms after the onset of the second sound. However, we did not observe any significant differences between different loudness percepts in gesture AWAY: the ERPs of “louder” perceptive shifts and no perceptive shifts did not differ (Figure 5F). The response magnitudes of N100 component were not significantly different either [*t*(16) = 0.17, *p* = 0.87, *d*_*z*_ = 0.04] (Figure 5G).

In general, these results, together with EE1, supported that the perceived loudness changes induced by the gesture CLOSER were consistently reflected in neurological measures as increases in early auditory ERP responses.

## Discussion

Our results from two behavioral experiments and two EEG experiments consistently demonstrate that visual-motor information in gestures can modulate the perception of a low-level auditory perceptual attribute such as loudness at the JND threshold. In BE1 and BE2, we found that gestures affected the judgment of loudness in accordance with their moving directions. In addition to the final position of hands, the visual-motor information exhibited extra influence on the loudness perception. The behavioral results in two EEG experiments replicated BE1. More importantly, in EE1, we found that the early neural responses to the sound stimuli were differently modulated by two gestures. In EE2, we found that biased judgments of loudness perception induced by gesture CLOSER showed larger N100 responses than unbiased judgments to the same stimuli. These consistent results collaboratively suggest that loudness perception can be modulated by the informational contents in other modalities that do not necessarily relate to auditory perception.

In BE1, we found that motional gestures modulated and interacted with the judgment of loudness change. Using the still CONST gesture as baseline conditions, we found that the AWAY gesture pushed participants’ judgments toward louder across all intensities, whereas the CLOSER gesture had the opposite effect – it pulled the judgments toward softer (Figure 2A). This tendency was also observed in the accuracy of judgments (Figure 2B). Specifically, accuracy was highest when the intensity changes of −1, 0, and +1 dB were paired with CLOSER, CONST, and AWAY gestures, respectively. The bias ratio further characterized the direction and the extent to which gestures biased the judgment of loudness under specific intensity changes (Figure 2C). We found that gestural directions biased the judgment of loudness when they were inconsistent. Interestingly, for +1 dB intensity change, CLOSER biased the responses off two levels (choosing “softer”) for about 40% of all the misjudgments made under that condition, and it was vice versa for −1 dB paired with AWAY. This effect was surprisingly big. One might argue that the 1 dB intensity change is hard to detect because it is close to the threshold, and participants might make their decision solely based on gestures. However, this was less likely given that the participants’ accuracy in the training sessions was above chance level (Supplementary Figure 1). Moreover, we explicitly told participants that the paring between sounds and gestures was completely random so that they should judge the sound intensity change only by what they heard.

We further probed the modulation effects of still gestures in BE2 to dissociate the factors of the distance between hands from the moving trajectories of gestures. We found that although both types of gestural stimuli had similar overall effects, the still gestures (SHORT, LONG) had a weaker influence on the judgment of loudness change (Figure 3A) than their motional versions (CLOSER, AWAY in BE1). Specifically, still gestures did not induce any significant effects on the judgment accuracy in most of the intensity change conditions (Figure 3B). Our findings suggest that the visual-motor information of gestures modulated the judgment of loudness differently from the spatial location between hands. This was in line with an fMRI study (Calvert and Campbell, 2003) reporting that moving speaking faces activated the auditory cortex and STS greater than still speech face images did. More importantly, our findings further suggested that motional gestures affected the judgment of loudness change not just by effects like a psychological suggestion or priming. Otherwise, the motional gestures would have very similar effects to still gestures.

EE1 was designed and analyzed in a “stimulus” perspective to investigate the nature of the observed modulation effects. That is, we examined whether and how the two motional gestures, CLOSER and AWAY modulated the early auditory neural responses. We identified an early neural modulation effect at around 110 ms (Figure 4D). Surprisingly, the sound stimuli induced stronger responses at around 110-ms latency when CLOSER rather than AWAY gesture was presented. The observed effect was not due to differences in visual responses to gestures because CLOSER and AWAY elicited similar visual neural responses in the time range of interest (Figure 4E). We did not observe such a pattern of stronger early auditory responses to lower intensity sounds when participants saw a blank screen or a still image (Supplementary Figure 3A) throughout the trial. Therefore, this ERP pattern we found in the AV conditions was specific to gestural modulation.

To provide further and stronger evidence, we carried out EE2 that tackled the same question in EE1 but from a complementary “perception” angle. We compared neural responses to the same auditory stimuli of no intensity change across two instances but with different loudness judgments to the second sound. In this case, the physical stimuli in each comparison were identical. The only difference was the participants’ loudness judgments. We found that the N100 ERP response was stronger when participants were biased by the CLOSER gesture to choose “second sound softer” than that when they were not perceptively biased. However, no significant neural modulation effect was found when participants were biased by AWAY gesture to choose “second sound louder.” This modulation pattern was consistent with the observation in EE1. It is worth mentioning that both EE1 and EE2 replicated the behavioral results of BE1. Crucially, EE2 ruled out the possibility that the observed modulation effects were caused by task demand, context, and stimuli in the specific experimental procedures. The finding strongly suggested that the biased judgments of loudness induced by gesture CLOSER were perceptual in nature.

In EE2, we found significant effects of CLOSER but not AWAY gesture on modulating auditory neural responses. These surprising but consistent asymmetric results could root in the inherent properties of auditory perception. Asymmetry of loudness perception and neural responses has been reported in various auditory tasks, such as auditory habituation (Butler, 1968), loudness recalibration (Marks, 1994; Mapes-Riordan and Yost, 1999), loudness adaptation (Canévet et al., 1985), and changing-loudness after effect (Reinhardt-Rutland, 2004). Interestingly, the changing-loudness after effect can also be induced if participants adapted to visual changing-depth, e.g., a box expanding or shrinking (Kitagawa and Ichihara, 2002). These studies suggest that asymmetry could indeed be a property in some forms of auditory perception. The modulation effects of gesture on loudness perception could also be asymmetrical so that the modulated responses associated with AWAY are smaller than the threshold that could be detected. Regardless of the asymmetry, the observations of modulation effects in early auditory responses support the hypothesis that visual-motor information in gestures can influence loudness perception.

The audiovisual paradigm we used introduces a challenge for the ERP analysis – the leading visual display induces visual responses that may temporally overlap with the subsequent auditory responses. In fact, we did not observe a clear auditory N1 component in EE1. This may be because the SOA of stimuli in different modalities was too short. Therefore, we used a longer SOA (230 ms) in EE2, which would minimize potential overlaps between the early auditory responses and visual responses to the preceding visual stimuli. The audiovisual integration likely occurs in a rather wide time window. So, the modulation effect would still be observed.

How loudness is represented in the brain is still unclear. Neuroimaging studies suggest that a full representation of perceived loudness completes at the cortical rather than the subcortical level (Röhl and Uppenkamp, 2012). According to electrophysiological studies (Thwaites et al., 2016) that analyzed the relations between EEG/MEG signals and loudness perception in different duration stimuli, the transformation of instantaneous loudness took place at 45- to 165-ms latency in Heschl’s gyrus and dorsal lateral sulcus. The cortical loudness representation (short-term loudness) can form as early as 45 ms in Heschl’s gyrus. Another transformation of the short-term loudness took place at 165- to 275-ms latency, such as at the length of a typical auditory word, in both dorsal lateral sulcus and superior temporal sulcus. We observed the modulation effect of gestures on loudness perception to simple vowels (/a/in our experiment) around 110 ms. The latency of the effect fell between the windows characterizing instantaneous loudness and the following possible transformation. Our results are consistent with previous literature about the dynamics of loudness perception and suggest that the perception of loudness might “superimpose” on auditory stimuli of different contents at different latencies.

Loudness perception is sensitive to context. Many studies have reported that loudness perception could be influenced by preceding sounds (Butler, 1968; Canévet et al., 1985; Näätänen and Picton, 1987; Lu et al., 1992; Marks, 1994; Mapes-Riordan and Yost, 1999; Näätänen and Winkler, 1999; Schmidt et al., 2020). At the neural population level, the dynamic range of auditory neurons of mammals could adapt to the intensity statistics of preceding sounds within a few seconds – a phenomenon called dynamic range adaptation (DRA). Evidence suggests that DRA first occurs in the auditory periphery (Wen et al., 2009) and develops along the auditory pathway, including the inferior colliculus (Dean et al., 2005) and the primary auditory cortex (Watkins and Barbour, 2008). Although different mechanisms have been proposed to account for various contextual effects, a common assumption is that loudness might be represented as relativity in the brain. In other words, what has been encoded is the change from a previous level instead of absolute magnitude. Our findings fit this view. We did not observe a simple relation among the sound intensity, loudness judgment, and ERP responses. In contrast, we observed larger N1 ERP responses to the sound with gesture CLOSER than with gesture AWAY in EE1. Moreover, the trials with “soft” bias evoked by CLOSER also showed a larger N1 component than trials with no bias in EE2. Such neural modulation effects were most likely reflecting the degree of loudness change. Moreover, we observed that the visual-motor information in gestures could influence auditory responses of loudness perception. This cross-modulation effect further suggests that the loudness perception is relative rather than directly linked to the absolute magnitude of physical, auditory stimuli.

Our results of cross-modulation on loudness perception are consistent with the framework of multisensory integration with some detailed exceptions. Audiovisual integration occurs in distributive cortices in various stages, with the most stable early effects around 100 ms after the sound onset (Talsma, 2015). The observed cross-modulation on loudness perception agrees with the timing of multisensory integration. Some theories assume the integration as unsupervised and bottom-up by combining information in two modalities based on spatial and temporal proximity (Alais and Burr, 2004; Baart et al., 2014). On the other hand, the multisensory integration could base on temporal predictions (van Wassenhove et al., 2005; Arnal et al., 2009) or predictions about features and categories (van Laarhoven et al., 2017). Moreover, iconic gestures and vocalization are innately connected in humans (Perlman and Lupyan, 2018). Our findings suggest that the early auditory responses reflect the modulation of gestures on loudness perception. However, the two gestures did not differ in their predictability or other aspects such as congruency or attention. The only difference was the moving direction that was remotely linked to loudness perception. Therefore, our results imply that factors other than predictability are likely to influence the amplitudes of early neural responses mediating loudness perception.

Manual gestures and speech have long been thought of as being integrated at the semantic and lexical level, with a few pieces of evidence suggesting a lower-level perceptual and productive connection. For example, observing motor acts of hand grasp modulated syllable pronunciation (Gentilucci, 2003). The lip aperture, voice peak amplitude and F0 frequency were greater when the observed hand grasp was directed to the large object. Our findings also suggest that gesture could modulate loudness perception, an attribute linking low-level features of speech perception. However, whether such modulation is due to a specific gesture-speech interaction or a general audiovisual interaction requires further investigation. It would be informative to probe the modulation effect by replacing the manual gestures with non-biological moving objects such as dots and bars, as well as extending to a wider range of featural differences.

The integrity of the gesture-speech system was often disrupted in various types of motor and psychopathological disorders, such as stuttering (Mayberry and Jaques, 2000), schizophrenia (Nagels et al., 2019), and autism spectrum (Silverman et al., 2010). Notably, tests based on sensory dominance and multisensory integration have been proposed as effective tools for the diagnosis of mild cognitive impairment in the elderly population (Murray et al., 2018). The findings in the current study may contribute to the development of new screening tools for psychopathological disorders involve the degradation of low-level multisensory processing.

The neural mechanisms that mediate multisensory integration, in particular the observed modulation effects of gestures on loudness perception, necessitate further investigation. Evidence suggests direct neural pathways between visual and auditory areas (Cappe and Barone, 2005). Silent movie clips of lip-movement activated auditory areas (Calvert et al., 1997; Calvert and Campbell, 2003; Besle et al., 2008). The earliest audiovisual cortical interaction can appear as early as 30 ms before the activation of polymodal areas (Besle et al., 2008). These studies indicate that cross-modal interaction could occur in a direct way between visual and auditory systems. Another possibility is that the interaction is mediated by the motor system. The motional gesture videos started 200 ms before the sound. The motion of gestures may induce corresponding motor representations that, in turn, transfer to sensory representations *via* the internal forward models (Tian and Poeppel, 2010, 2012). These sensory representations may share some common representational features that might be much easier to integrate with the processing of external auditory stimuli (Tian et al., 2018; Zhen et al., 2019). For loudness perception, the converted distance and speed information from the motor system may have an abstract representation for magnitude that can interact with the rate coding of loudness perception (Glasberg and Moore, 2002; Röhl and Uppenkamp, 2012; Thwaites et al., 2016).

In conclusion, we found that motional gestures influenced the judgment of loudness change at the JND threshold. Moreover, the cross-modal effects on loudness perception were temporally localized in the early auditory neural responses. The consistent results in four behavioral and EEG experiments suggest that gestures can modulate loudness perception. These findings provide evidence suggesting that visual-motor events can penetrate the processes of primary perceptual attributes in auditory perception.

## Data Availability Statement

The data will be available upon request to interested researchers.

## Ethics Statement

The studies involving human participants were reviewed and approved by local Research Ethics Committee at NYU Shanghai. The patients/participants provided their written informed consent to participate in this study.

## Author Contributions

XT, JS, and ZW conceived the study. JS and XT wrote the manuscript. JS, ZW, and XT designed the experiments. JS and ZW carried out the experiments and pre-processed the EEG data. JS analyzed the data and made the tables and figures. All authors contributed to the article and approved the submitted version.

## Conflict of Interest

The authors declare that the research was conducted in the absence of any commercial or financial relationships that could be construed as a potential conflict of interest.

## Publisher’s Note

All claims expressed in this article are solely those of the authors and do not necessarily represent those of their affiliated organizations, or those of the publisher, the editors and the reviewers. Any product that may be evaluated in this article, or claim that may be made by its manufacturer, is not guaranteed or endorsed by the publisher.
