# Supplementary material for: Manual Gestures Modulate Early Neural Responses in Loudness Perception
Source: Front Neurosci. 2021 Sep 1;15:634967. doi: 10.3389/fnins.2021.634967 (PMC8440995; doi:10.3389/fnins.2021.634967)
Supplement: Supplementary file 1 [file Data_Sheet_1.pdf]

Supplementary Table 1. The setup of experiment conditions in EE1

Arrangement	1st part of a block in the main experiment											
Category	AV						A					
Gesture	CLOSER			AWAY			CONST			BLANK		
Intensity change	-1 dB	0 dB	+1 dB	-1 dB	0 dB	+1 dB	-1 dB	0 dB	+1 dB	-1 dB	0 dB	+1 dB
Number of trials per block	4	4	4	4	4	4	4	4	4	4	4	4
Total number of trials	48	48	48	48	48	48	48	48	48	48	48	48

Arrangement	2nd part of a block in the main experiment	
Category	V	
Gesture	CLOSER	AWAY
Number of trials per block	6	6
Total number of trials	72	72

Arrangement	after the main experiment	
Category	intensity localizer	
Intensity	low intensity	high intensity
Number of trials per block	70	70
Total number of trials	70	70

Supplementary Table 2. The confusion matrix table for the four experiments.

	gesture	intensity change (dB)	judgment (%)		
			softer	unchanged	louder
BE1	CLOSER	-1	47.8	40.1	12.1
		0	36.1	42.1	21.8
		1	27.0	40.4	32.5
	CONST	-1	28.0	59.5	12.5
		0	17.0	61.4	21.6
		1	10.8	56.3	32.9
	AWAY	-1	24.5	39.9	35.7
		0	14.6	39.7	45.7
		1	10.3	33.9	55.9
BE2	SMALL	-1	48.0	40.5	11.6
		0	26.8	55.7	17.5
		1	15.5	45.4	39.1
	MIDDLE	-1	39.0	50.0	11.0
		0	19.0	60.7	20.2
		1	7.2	46.2	46.6
	LARGE	-1	41.7	42.1	16.3
		0	17.9	46.2	35.9
		1	7.7	30.4	61.9
	NO GESTURE	-1	40.9	48.8	10.3
		0	20.3	60.4	19.3
		1	9.6	51.7	38.7
EE1	CLOSER	-1	69.2	24.4	6.4
		0	27.6	53.3	19.1
		1	17.1	31.9	51.0
	CONST	-1	48.5	44.0	7.5
		0	13.8	73.4	12.8
		1	7.7	54.5	37.8
	AWAY	-1	49.2	28.9	21.9
		0	10.5	40.1	49.4
		1	5.3	16.9	77.7
	BLANK	-1	50.3	43.5	6.3
		0	13.1	69.3	17.6
		1	6.9	48.9	44.2
EE2	CLOSER	-1	58.6	32.8	8.6
		0	38.4	50.4	11.3
		1	28.7	46.7	24.5
	AWAY	-1	32.9	28.9	38.2
		0	14.5	38.6	46.9
		1	8.2	30.6	61.2

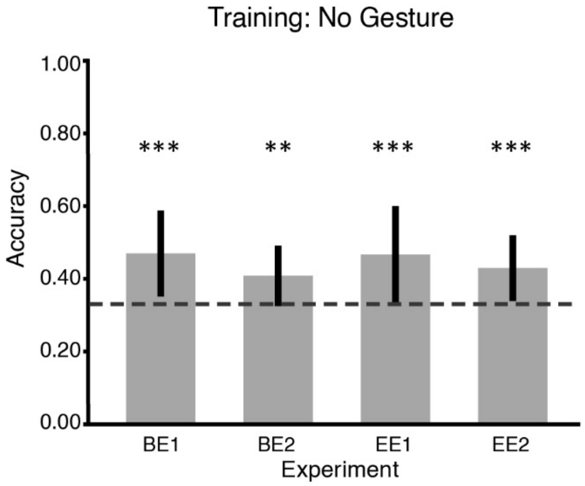

Supplementary Figure 1. The accuracy in the training session of BE1, BE2, EE1, and EE2. Accuracy in judging the intensity change in the absence of gesture was above chance level (0.33, indicated as a dashed line in the figure) in all experiments. The error bars indicate ± one standard deviation. **p* <0.05; ** *p* <0.01; *** *p* <0.001

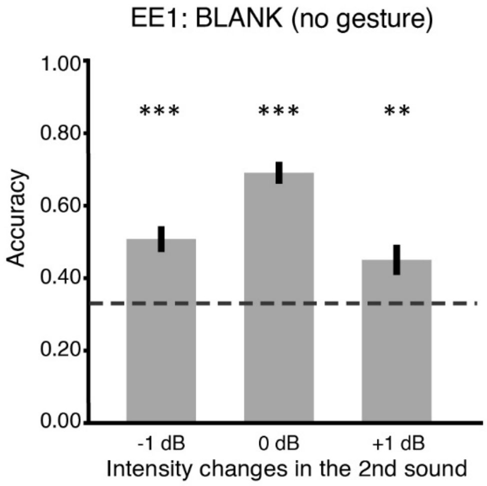

Supplementary Figure 2. Accuracy of the behavioral judgment about intensity change in the BLANK (no-gesture) condition of EE1. The dashed line indicates the chance level (0.33). All error bars indicate ± one standard error of the mean (SEM). **p*<0.05; ***p* <0.01; ****p* <0.001

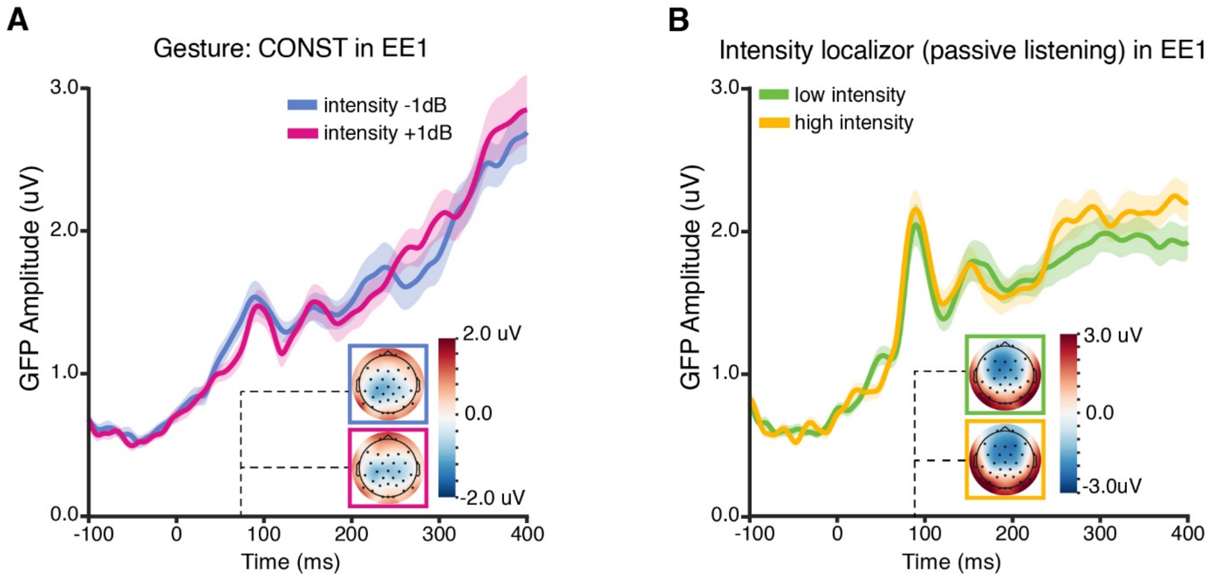

Supplementary Figure 3. ERP waveforms and topographies in auditory control conditions in EE1. **(A)** ERP responses to auditory stimuli in the CONST conditions where the sounds were presented while a still image of gesture stayed on the screen. No significant difference between the two conditions has been revealed by temporal cluster analysis. **(B)** ERP responses to the intensity localizers in which participants passively listened to a sequence of pure tones with two levels of intensity without any visual stimuli. No significant difference between the two conditions has been revealed by temporal cluster analysis. The solid lines indicate the grand mean global field power (GFP) waveforms. The shades around the solid lines represent $\pm$ one SEM. Response topographies are shown in colored boxes with dashed lines pointing to their latencies. The colored boxes use the same color schemes as the waveform responses to indicate different conditions.

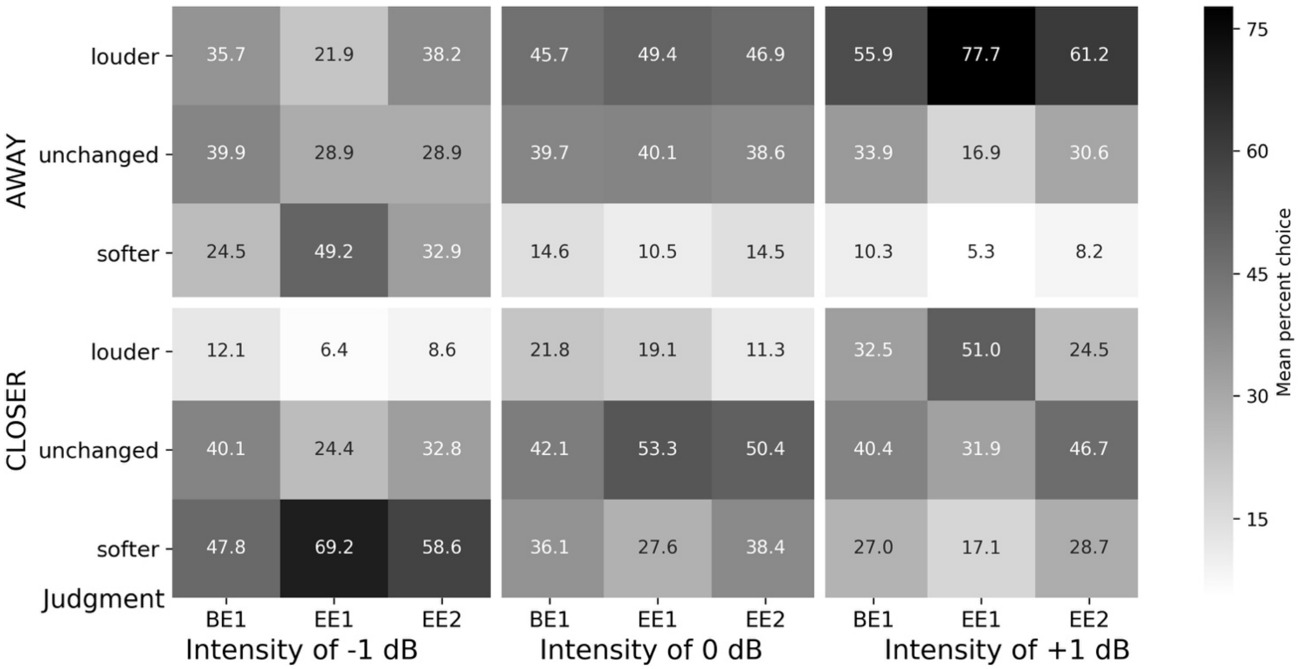

Supplementary Figure 4. Confusion matrix for the two motion gestures across BE1, EE1, and EE2. They are a subset of Supplementary Table 1**.** The numbers in the confusion matrices represent the percent choice averaged across participants for each gesture-intensity pair in each experiment. The three values in any of the three consecutive vertical boxes add up to 100. For each 3-by-3 box in the figure, the y-axis indicates the behavioral judgment on the loudness change to the second sound, while the x-axis indicates the three experiments using motional gestures. In each of the three intensity-change conditions (three columns), the judgment patterns under gesture AWAY (upper rows) are relatively skewed towards 'louder' compared with the judgment pattern under CLOSER (lower rows).
